# Strain-Dependent Restriction of Human Cytomegalovirus by Zinc Finger Antiviral Proteins

**DOI:** 10.1128/jvi.01846-22

**Published:** 2023-03-14

**Authors:** Maria Jose Lista, Adam A Witney, Jenna Nichols, Andrew J Davison, Harry Wilson, Katie A Latham, Benjamin J Ravenhill, Katie Nightingale, Richard J Stanton, Michael P Weekes, Stuart J D Neil, Chad M Swanson, Blair L Strang

**Affiliations:** a Department of Infectious Diseases, School of Immunology & Microbial Sciences, King’s College London, London, UK; b Institute of Infection & Immunity, St George’s, University of London, London, UK; c MRC-University of Glasgow Centre for Virus Research, Glasgow, UK; d Cambridge Institute for Medical Research, School of Clinical Medicine, University of Cambridge, Cambridge, UK; e Division of Infection and Immunity, Cardiff University School of Medicine, Cardiff, UK

## Abstract

Cellular anti-viral factors that recognize viral nucleic acid can inhibit virus replication. These include the zinc finger antiviral protein (ZAP), which recognizes high CpG dinucleotide content in viral RNA. Here we investigated the ability of ZAP to inhibit the replication of human cytomegalovirus (HCMV). Depletion of ZAP or its co-factor KHNYN increased the titre of the high passage HCMV strain AD169, but had little effect on the titre of the low passage strain Merlin. We found no obvious difference in expression of several viral proteins between AD169 and Merlin in ZAP knockdown cells, but observed a larger increase in infectious virus in AD169 compared to Merlin in the absence of ZAP, suggesting that ZAP inhibited events late in AD169 replication. In addition, there was no clear difference in the CpG abundance of AD169 and Merlin RNAs, indicating that genomic content of the two virus strains was unlikely to be responsible for differences in their sensitivity to ZAP. Instead, we observed less ZAP expression in Merlin infected cells late in replication compared to AD169 infected cells, which may be related to different abilities of the two virus strains to regulate interferon signaling. Therefore, there are strain-dependent differences in the sensitivity of HCMV to ZAP, and the ability of low passage HCMV strain Merlin to evade inhibition by ZAP is likely related to its ability to regulate interferon signaling, not the CpG content of RNAs produced from its genome.

## Introduction

A number of intracellular proteins are dedicated to detecting viral RNA as a defense mechanism, including the zinc finger antiviral protein (ZAP, also known as PARP13 or ZC3HAV1) (1). This protein recognizes viral RNA genomes due to the unusually high proportion of CpG dinucleotides present in viral RNA in comparison with most cellular transcripts (2–4). Although a putative ZAP recognition sequence containing a CpG dinucleotide in RNA has been described (5), increasing evidence indicates that ZAP recognition of RNA is dependent on the abundance and location of CpG dinucleotides in RNA (6–8). The presence of U or A nucleotides in CpG-rich RNA (8) or a high UpA dinucleotide content in RNA (9, 10) may also regulate ZAP binding. Recognition of viral RNA by ZAP or inhibition of translation by ZAP can lead to viral RNA degradation (11–15).

Different isoforms of ZAP can be expressed within an infected cell. The long isoform, ZAP-L, is a constitutively expressed cytoplasmic protein that is associated with cytoplasmic membranes due to a S-farnesylation post-translational modification that requires the C-terminal CaaX box (16). The short isoform, ZAP-S, is produced upon activation of the type I interferon system (17) by alternative splicing and polyadenylation (18–20). Therefore, both ZAP-L and ZAP-S share a common amino-terminus, which includes four zinc finger domains that mediate RNA bindng (21). As ZAP-S does not share a carboxy-terminus with ZAP-L, it does not contain either the catalytically inactive ADP-ribosyltransferase (ART) domain or the CaaX box that is present in the carboxy-terminus of ZAP-L (16, 19).

ZAP-L and ZAP-S inhibit the replication of RNA viruses from diverse families, including retroviruses, alphaviruses, filoviruses, flaviviruses and picornaviruses (3, 11, 15, 22–25), but it is unclear or unknown why either or both ZAP-L and ZAP-S proteins have inhibitory effects against some viruses and not others (11, 15, 22–25).

ZAP alone does not have the ability to degrade RNA, which is likely to be mediated by associated cellular nucleases. These nucleases include the recently discovered ZAP co-factor KHNYN, a putative endoribonuclease that is able to interact with both ZAP-L and ZAP-S (26). Another ZAP co-factor is the ubiquitin E3-ligase TRIM25, the presence of which is required for ZAP function during inhibition of several viruses, for example Sindbis virus and retroviruses (27, 28). It is unclear whether TRIM25 is required for ZAP function against all viruses and how TRIM25 modulates the antiviral activity of ZAP is unknown.

To date, most studies have focused on the interaction of ZAP and its co-factors with RNA virus genomes. However, there is increasing evidence that ZAP inhibits the replication of viruses with DNA genomes, possibly by recognizing viral RNAs with high CpG dinucleotide content transcribed from viral DNA. Moreover, there is strong evidence that DNA viruses can evade ZAP function by expressing proteins that can antagonize ZAP. Vaccinia virus (strain Copenhagen) evades ZAP function through the viral protein C16, which sequesters ZAP in cytoplasmic punctate structures (29). Herpes simplex virus (HSV) endonuclease vhs mediates degradation of the mRNA encoding ZAP proteins (30) and the murine gammaherpes virus 68 protein RTA inhibits dimeric ZAP interaction (21, 31).

Recently, ZAP restriction of another herpesvirus, human cytomegalovirus (HCMV), was investigated (32). It was proposed that either ZAP-L or ZAP-S as well as TRIM25 inhibited replication of the HCMV strain TB40/E via recognition of high CpG dinucleotide content in the viral mRNA encoding HCMV proteins essential for virus replication (32). A further study (33) using HCMV strain TB40/E also demonstrated ZAP proteins could prevent production of HCMV strain TB40/E from infected cells and indicated that ZAP could destabilize expression of certain HCMV mRNAs, although the functional relevance of those observations remains unclear.

HCMV strain TB40/E genome organization is similar to that of low passage HCMV strains, whose genomes are similar to clinical HCMV strains present in patients (34). However, the TB40/E genome also contains many mutations across the genome that differ from clinical strains (34–38). Thus, it was possible that clinical HCMV strains carry additional functions to evade ZAP restriction, enabling more efficient replication *in vivo*. This could help explain how clinical HCMV strains would evade restriction by ZAP to replicate efficiently in humans. To better understand ZAP restriction of HCMV more fully, we compared the ability of ZAP to restrict replication of the well characterized high passage HCMV strain AD169 and an HCMV strain (Merlin) which has a genome nearly identical to that found *in vivo*. We found strain-dependent differences in the inhibitory ability of ZAP. This difference in restriction between these strains was not obviously related to the CpG dinucleotide content of RNAs expressed by their genomes or differences in viral protein expression, but instead correlated with a ZAP-dependent inhibition of infectious virus production.

## Materials & Methods

### Cells and viruses

Human foreskin fibroblast (HFF) cells (clone Hs27) were obtained from American Type Culture Collection no. CRL-1634 (ATCC, Manassas, VA). All cells were maintained in complete media: Dulbecco’s Modified Eagle’s Medium (DMEM) (Gibco) containing 5% (v/v) fetal bovine serum (FBS) (Gibco), plus 1% penicillin-streptomycin (Invitrogen). HCMV strain AD169 was a gift from Donald Coen (Harvard Medical School), and HCMV strain TB40/E was generated from a bacmid containing the TB40/E genome (39) and generously provided by Matthew Reeves (University College London). The generation of HCMV strain Merlin(R1111) from a bacmid that contains engineered mutations in genes RL13 and UL128 to allow release of cell-free virus has been reported elsewhere (40). Engineering of reporter viruses HCMV strain Merlin(R1278) and Merlin(R1293) from Merlin(R1111) encoding bacmids to contain a reporter gene cassette and specific mutations within the Merlin genome has also been reported elsewhere (41). Sendai virus (SeV) strain Cantell in amnioallantoic fluid was the kind gift of Steve Goodbourn (St George’s, University of London).

### Lentivirus treatment of HFF cells

Design and generation of HIV vectors carrying LentiCRISPR genomes encoding guide RNAs targeting RNA encoding Luciferase (Luc), β-galactosidase (LacZ), ZAP, KHNYN, and TRIM25, plus a puromycin resistance gene, have been described elsewhere (26). For ZAP the guide previously described as ZAP-G1 (26), which recognises sequence in ZAP exon 6 (present in mRNA encoding all known ZAP proteins) was used. For KHNYN, the guide previously described as KHNYN-G1 (26) was used. HFF cells were infected with HIV vectors carrying each of the LentiCRISPR genomes at a multiplicity of infection (MOI) of approximately 0.5. After incubation for 48 h, the cells were were incubated in medium containing 0.5 μg/ml puromycin for 24 h. The puromycin-resistant cells were then used for the experiments, described in the text and Figures, being maintained by further incubation with puromycin every 4-6 passages.

### Western blotting

Lysate of uninfected or infected HFF cells were prepared for western blotting by washing the cells once in phosphate-buffered saline (Gibco; PBS), suspending the cells directly in 2 x Laemmli buffer containing 5% β–mercaptoethanol, and incubating at 95oC for 5 min. Immunoblotting of proteins separated on 8% or 10% (v/v) polyacrylamide gels was carried out using antibodies recognizing HCMV IE1/2, UL44, pp65 or pp28, (all Virusys, 1:1000 dilution), β-actin (SIGMA, 1:5000 dilution), HCMV UL86 (a kind gift from Wade Gibson, Johns Hopkins University, 1:5000 dilution), TRIM25 (Abcam, ab167154, 1:5000 dilution), ZAP (Abcam, ab154680, 1:5000 dilution), KHNYN (Santa Cruz Biotech, sc-514168, E-3 clone 1:100 dilution), IRF-3 (Cell Signaling, 4302, clone D83B9, 1:1000 dilution) and Phospho-IRF-3 (Ser396) (Cell Signaling, 4947, clone 4D4G, 1:1000 dilution). All primary antibodies were incubated overnight at 4°C and detected using anti-mouse- or anti-rabbit-horseradish peroxidase (HRP) conjugated antibodies (Millipore and Cell Signaling Technologies, respectively), except for KHNYN detection where anti-mouse IgG HRP-linked (Cell Signaling Technologies) was used. All incubation steps were carried out using 5% powdered milk in TBS-T, except for Phospho-IRF-3 where 5% bovine serum albumin in TBS-T was used. Chemiluminescence solution (GE Healthcare) were used to detect secondary antibodies. Chemiluminescence signal was recorded on X-ray film (MOL7016, SLS), except for KHNYN detection which was visualized using an ImageQuant 800 (Amersham) apparatus. If necessary blots were stripped and re-probed as described above. Relative band intensity (band intensity relative to β-actin signal in the same lane) was analyzed using ImageJ software, obtained from the NIH (USA).

### Determination of viral titer by virus titration

Titers were determined by serial dilution of viral supernatant onto HFF monolayers, which were then covered in DMEM containing 5% (v/v) FBS, 1% penicillin-streptomycin (Invitrogen) and 0.6% (w/v) methylcellulose. After incubation for 14 days, cells were stained with crystal violet and plaques in the infected cell monolayers were counted. Titre was expressed as plaque-forming units (p.f.u.)/ml.

### Transfection of siRNA into HFF cells

Briefly, 1 x 10^5^ HFF per well were seeded in 12-well plates 24 hours before transfection in DMEM+5%FBS with no antibiotics. Per well, 113 µl of 1 µM siRNA and 2 µl Dharmafect2 (Dharmacon) were diluted in 93 µl and 146 µl Optimem (Invitrogen), respectively. After 5 mins at room temperature, both solutions were combined. After 20 mins, media was removed from each well and replaced with the siRNA/Dharmafect mixture and 500 µl of DMEM+5%FBS with no antibiotics was added to each well. Transfected cells were incubated at 37°C for 72 hours then either prepared for western blotting or infected with 1 x 10^5^ plaque forming units (p.f.u./ml) of AD169. Double stranded siRNA targeting expression of either ZAP-L or ZAP-S (and a non-targeting control double stranded siRNA) have been described elsewhere (20). siRNA targeting ZAP-L expression binds RNA in exon 12 of mRNA encoding ZAP-L, which is not present in mRNA encoding ZAP-S. siRNA targeting ZAP-S expression binds the 3’UTR of mRNA encoding ZAP-S, which is not present in mRNA encoding ZAP-L (20).

### Interferon and Ruxolitinib

Interferon-α and MG132 were kind gifts from Steve Goodbourn (St George’s, University of London). Ruxolitinib (Cambridge Bioscience) and MG132 were resuspended in dimethyl sulfoxide (DMSO). Unless stated otherwise, cells were treated with 1000U/ml of Interferon-α (or the equivalent volume of cell culture medium) or 10 μM Ruxolitinib or 10 μM MG132 (or the equivalent volume of DMSO).

### Quantitative analysis of RNA expression

RNA from cells was extracted using Qiagen RNeasy (Qiagen RNeasy minikit; 74106) following the manufacturer’s instructions. One µg of purified RNA from each extraction was reversed transcribed using High-Capacity cDNA Reverse Transcription Kit (Applied Biosystems; 4368814). Quantitative PCR was performed using TaqMan™ Universal PCR Master Mix (Applied Biosystems; 4304437). The relative abundance of ZAP in each sample was measured using ZAP Taqman Assay (Applied Biosystems; Hs00912660_m1) and normalized to GAPDH levels using GAPDH Taqman Assay (Applied Biosystems; Hs99999905_m1).

### Sequencing of HCMV genomes

HFF cells (1x10^5^) were infected at an MOI of 1 for 72 h. DNA was extracted from infected cells and sheared using a Covaris S220 to an approximate size of 450 bp. Sequencing libraries were created using a Kapa LTP Library preparation kit according to the manufacturer’s instructions, employing indexed primers (New England Biolabs) for PCR. The libraries were sequenced on a NextSeq Mid Output 300 cycle cartridge to produce approximately 5 million paired-end reads of 150 nucleotides (nt). Sequence data have been deposited in the European Nucleotide Archive (ENA) at EMBL-EBI under accession number PRJEB58764.

### Bioinformatics of HCMV genomes

Genome sequences for each virus strain were obtained from NCBI, AD169 (BK000394.5), Merlin (NC_006273.2), TB40/E clone TB40-BAC4 (EF999921.1). The observed versus expected (obs/exp) ratio was calculated for each gene as:

Obs/ExpCpG=NumberofCpG*N/(NumberofC*NumberofG)

where N is the length of the sequence.

### Preparation of virus from infected cell supernatant for western blotting

Cells were infected as described in the text and figure legend. At 96 h post-infection, viral supernatant was collected (9 ml in total) and clarified by centrifugation (13,000*g*, 5 min, 4°C) to remove cells and cell debris. Virions were then pelleted from the supernatant by ultracentrifugation (20,000 rpm for 1 h at 4°C) and resuspended in 20 μl PBS. Each resuspended pellet was incubated with 20 μl trypsin (Gibco) for 1 h at 37°C, supplemented with 20 μl 2x Laemmli buffer containing 5% (v/v) 2-mercaptoethanol, and incubated at 95°C for 5 min. Western blotting was carried out as outlined above.

### Preparation of virus associated with infected cells

HCMV Infections were carried out as described in the text and Figures. Cell-released virus in the infected cell supernatant was removed and titrated. To prepare cell-associated virus, the cells were scraped from the dish into 1 ml cell culture medium using a pipette tip and sonicated using a benchtop water bath sonicator (full power for 3 x 30 s), and the cell debris was pelleted by benchtop centrifugation (13,000 rpm for 5 min). An aliquot (500 µl) from the top of the supernatant was transferred to a new tube and titrated.

## Results

### Restriction of HCMV strain AD169, but not Merlin, by ZAP and KHNYN

**Figure 1:**
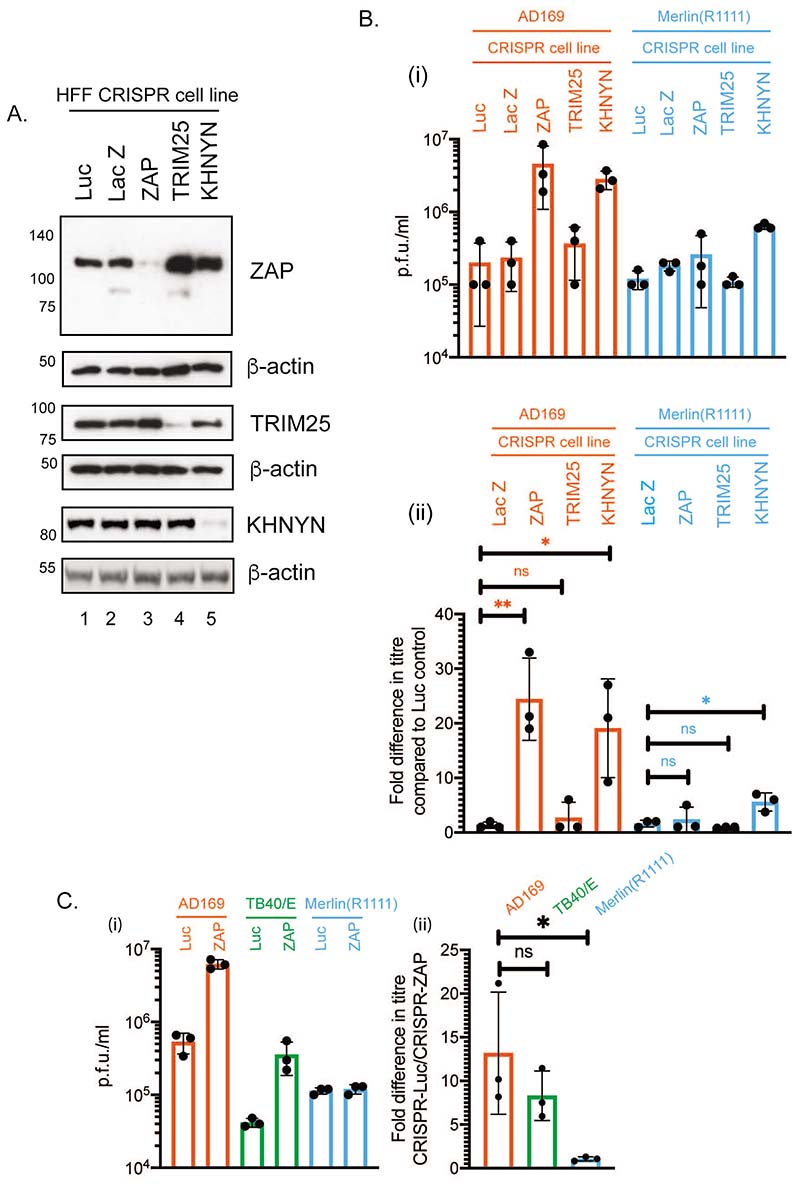
HCMV replication in HFF cells containing CRISPR. (A) Uninfected cell lysates were prepared for western blotting. Each CRISPR containing cell line used is indicated above the figure. Proteins recognized by the antibodies used in the experiment are indicated to the right of the figure. The positions of molecular weight markers (kDa) are indicated to the left of the figure. Relative band intensity (band intensity relative to β-actin signal in the same lane) was analyzed using ImageJ and fold knockdown of proteins compared to CRISPR-Luc is reported in the text. The data is representative of two independent experiments. (B and C) Cell lines shown in panel (A) were infected at a multiplicity of infection of 1. Virus in infected cell supernatant was collected at 96 hours post infection and viral titre was determined by titration of virus supernatant on HFF cells. Virus and cell line used is shown below each figure (i) Titre in plaque forming units/ml (p.f.u./ml) of each experiment. (ii) Fold increase in virus titre in each cell line compared to virus titre from HFF cells containing CRISPR inhibiting Luciferase expression. In each figure data is representative of three independent experiments (black data points) and presented as average (block) and standard deviation (error bars) of the data. Statistical relevance was examined used a student t test. ns = not significant (ns), p=<0.05 (*), p=<0.01 (**).

We wished to understand if ZAP and its co-factors, TRIM25 and KHNYN, could restrict HCMV replication. HFF cells were treated with lentiviruses expressing Cas9 and various CRISPR guide RNAs (gRNAs). CRISPR knockdown of protein expression was confirmed using western blotting (Fig. 1A) Relative band intensity was used to measure protein knockdown compared to CRISPR-Luc; ZAP knockdown of 9 fold (compare lanes 1 and 3), TRIM25 knockdown of 6 fold (compare lanes 1 and 4), KHNYN knockdown of 12.1 fold (compare lanes 1 and 5). These cells were infected with either the high passage HCMV strain AD169 or the clinical-like strain Merlin(R1111) (34). We compared the viral titre from either ZAP, TRIM25 or KHNYN depleted cells to cells containing CRISPR-Luc (Fig. 1B(i)). ZAP or KHNYN depletion resulted in a notable (over 20-fold) increase in AD169 titre (Fig. 1B(ii)). Loss of ZAP had no substantial effect on Merlin(R1111) titre, but loss of KHNYN resulted a modest (4-6 fold) increase in Merlin(R1111) titre (Fig. 1B(ii)). Therefore, ZAP could restrict production of high passage strain AD169, but not low passage strain Merlin(R1111) while KHNYN may have both ZAP-dependent and ZAP-independent effects on HCMV replication. Interestingly, TRIM25 depletion had little or no effect on titre of either AD169 or Merlin(R1111) viruses (Fig. 1B(ii)).

It had been reported that ZAP could restrict replication of HCMV strain TB40/E (32, 33). We confirmed this by demonstrating an increase in TB40/E titre from CRISPR-ZAP cells compared to CRISPR-Luc cells (Fig. 1C) This confirmed that there are HCMV strain-specific differences for ZAP sensitivity and infectious virus release of the primary isolate Merlin(R1111) was not restricted.

### ZAP-S contributes to HCMV restriction

**Figure 2:**
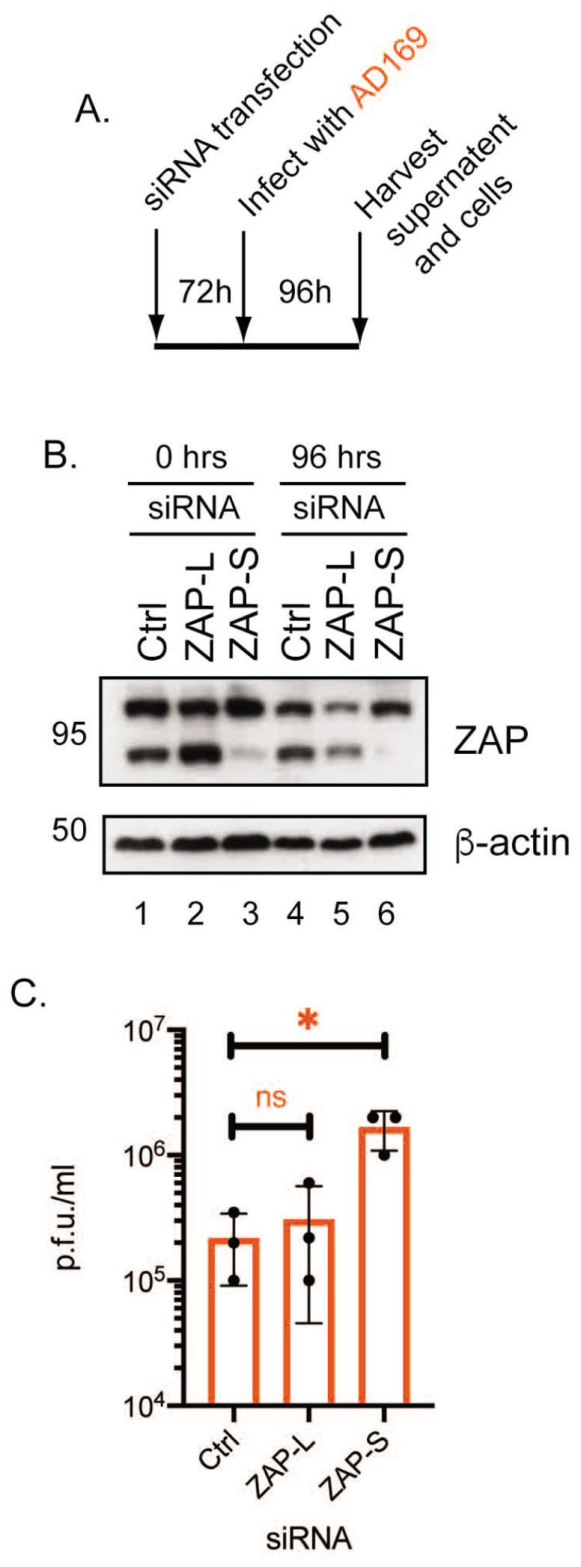
HCMV replication in HFF cells treated with siRNAs inhibiting ZAP expression. (A) Schematic of HFF cells were treated with Ctrl, ZAP-L siRNA or ZAP-S siRNAs infected with HCMV. At the time points indicated in the figure (hours post infection (h.p.i.)) viral supernatant was collected and lysates were prepared for western blotting from infected cells treated with siRNA. (B) Western blotting of cell lysates from uninfected and infected cells. Each condition used is shown above the figures. Proteins recognized by the antibodies used in each experiment are indicated to the right of each figure (ZAP-L (101 kDa) or ZAP-S (78 kDa)). The positions of molecular weight markers (kDa) are indicated to the left of each figure. Relative band intensity (band intensity relative to β-actin signal in the same lane) was analyzed using ImageJ and fold knockdown of proteins compared to the same protein in Ctrl siRNA treated cells is reported in the text. The data is representative of two independent experiments. (C) Viral titre of virus in cell supernatant harvested at 96 hours post infection from the infected cells analyzed in (B) determined by titration of virus supernatant on HFF cells. Data shown are the mean and standard deviations of data from three independent experiments. Viral titre is expressed at plaque forming units/ml (p.f.u./ml). The data is representative of three independent experiments (black data points) and presented as average (block) and standard deviation (error bars) of the data. Statistical relevance was examined used a student t test. ns = not significant (ns), p=<0.05 (*).

To determine whether ZAP-L or ZAP-S had inhibitory effects on HCMV replication HFF cells were treated with siRNA that inhibited expression of either ZAP-L or ZAP-S (20), or a non-targeting control siRNA (20). The siRNA treated HFF cells were then infected with AD169 (Fig. 2A). siRNA targeting ZAP-L had little effect on ZAP-L expression in uninfected and infected cells (2-fold knockdown compared to Ctrl siRNA, compare lanes 4 and 5 in Fig. 2B). Conversely, siRNA targeting ZAP-S caused a substantial decrease in ZAP-S expression (7-fold knockdown compared to Ctrl siRNA, compare lanes 4 and 6 in Fig. 2B) and no obvious effect on ZAP-L expression (Fig. 2B). The presence of siRNA targeting ZAP-L had no obvious effect on virus titre (Fig. 2C), most likely due to inefficient protein knockdown (Fig. 2B). Nevertheless, loss of ZAP-S expression resulted in an approximately 10-fold increase in AD169 titre (Fig. 2C). This indicated that not only could ZAP-S restrict HCMV replication, but the magnitude of restriction suggested that ZAP-S is an important inhibitory isoform of ZAP in HCMV infected cells. This is consistent with previous observations that ZAP-S was robustly expressed in TB40/E infected cells throughout HCMV replication (32, 33). However, it has been reported elsewhere that ZAP-L can inhibit HCMV protein production or replication (32, 33).

### ZAP expression differs between HCMV AD169 and Merlin infected cells

**Figure 3:**
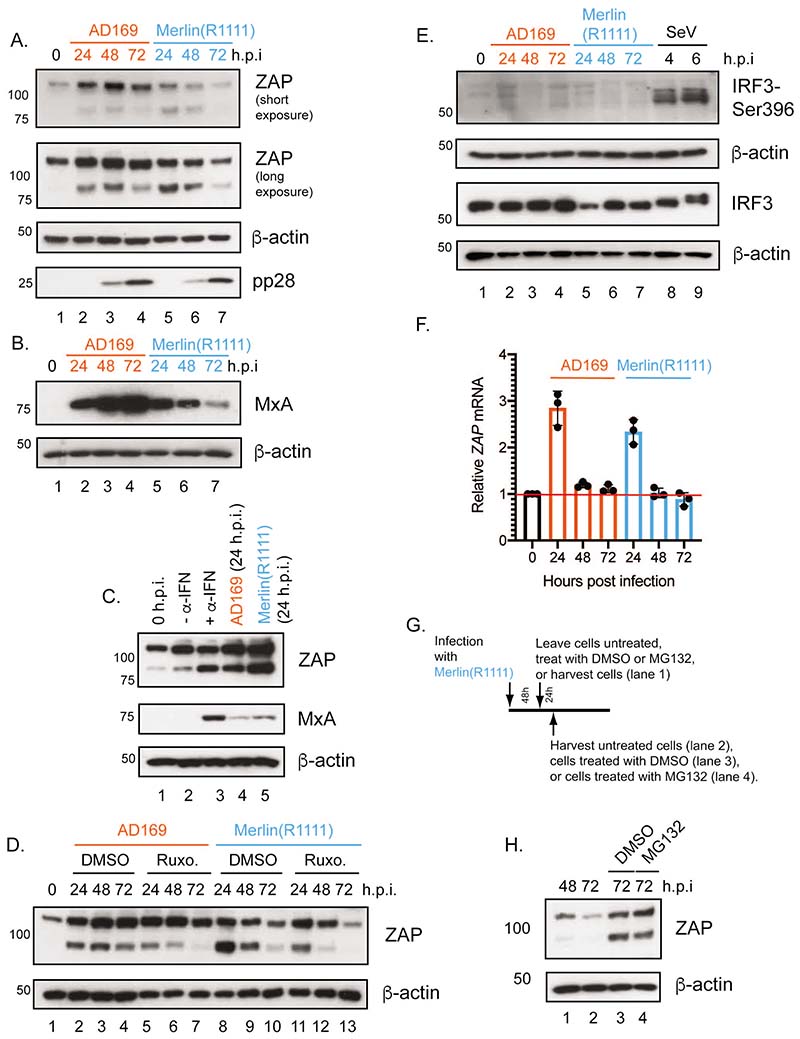
ZAP protein and RNA expression in HFF cells infected with AD169 or Merlin(R1111). (A and B) HFF cells were infected at a multiplicity of infection of 1 with either AD169 or Merlin(R1111). Cell lysates were prepared for western blotting at the time points (hours post infection (h.p.i.)) indicated above the figure. (C) Cell lysates were prepared for western blotting form uninfected cells at the time of treatment (0 h.p.i.), from HFF cells treated with complete media or complete media containing α-IFN (-α-IFN and +α-IFN, respectively) for 24 hours and from HFF cells infected with either AD169 or Merlin(R1111) (24 hours post infection). (D) HFF cells were infected at a multiplicity of infection of 1 with either AD169 or Merlin(R1111) and then treated with either DMSO or Ruxolitinib (Ruxo.). (E) HFF cells were infected with HCMV or Sendai virus (Cantell) at a multiplicity of infection of 1 or a dilution of 1:50, respectively. Cell lysates were prepared for western blotting at the time points (hours post infection (h.p.i.)) indicated above the figure. (F) HFF cells were infected at a multiplicity of infection of 1 with either AD169 or Merlin(R1111). Cells were prepared for preparation of RNA at the time points (hours post infection (h.p.i.)) indicated above the figure. RNA was prepared from cells and in each sample the number of copies of *ZAP* mRNA (encoding both ZAP-S and ZAP-L) and cellular *GAPDH* mRNA were assayed using quantitative PCR. For each reaction the 2ˆ(-deltaCT) value was calculated. Relative abundance of *ZAP* mRNA to *GAPDH* mRNA was calculated and values from infected cells were normalized to the values from uninfected cells. The data is representative of three independent experiments (black data points) and presented as average (block) and standard deviation (error bars) of the data. The red horizontal bar indicates a value of 1, the data from uninfected cells. (G and H) HFF cells were infected at a multiplicity of infection of 1 with Merlin(R1111). Infected cells were treated as shown in Figure G and described in the text. In Figure H cell lysates from the experiments described in Figure G were prepared for western blotting at the time points (hours post infection (h.p.i.)) indicated above the figure. In all western blotting figures proteins recognized by the antibodies used in each experiment are indicated to the right of each figure. The positions of molecular weight markers (kDa) are indicated to the left of each figure. In those experiments uninfected cells harvested at the time of infection are shown as 0 h.p.i..

Next, we examined ZAP expression in HCMV infected cells. We found less ZAP-L and ZAP-S expression in Merlin(R1111) infected cells at 48 and 72 hours post-infection (h.p.i.) compared to AD169 infected cells (Figs. 3A and S1). We did not observe a difference in expression of HCMV late protein pp28 in Merlin(R1111) and AD169 infected cells over time, suggesting similar levels of infection between the two HCMV strains (Fig. 3A). Less ZAP expression in Merlin(R1111) infected cells compared to AD169 infected cells was also found in experiments using CRISPR-Luc at 48 and 72 h.p.i. (Fig. S2A and S2B). Additionally, the absence or ZAP had no effect on either KHNYN or TRIM25 expression (Fig. S2C) and the absence of either KHNYN or TRIM25 had no obvious effect on ZAP protein expression in HCMV infected cells (Fig. S2A and S2B), although loss of TRIM25 appeared to increase ZAP-L expression (Fig. S2B). Therefore, lower ZAP expression in Merlin(R1111) infected cells may have contributed to the ability of Merlin(R1111) to avoid restriction by ZAP.

A decrease in ZAP expression over time in Merlin(R1111) infected cells was consistent with a previous study that found a decrease in ZAP expression in Merlin(R1111) infected cells over time using mass spectrometry (42). That study also demonstrated that many proteins expressed in response to type I interferon signaling, such as MxA, decreased over time in Merlin(R1111) infected cells (42). To confirm and extend these findings, we compared MxA expression between AD169 and Merlin(R1111) infected cells, and found that it was correlated with ZAP expression (Fig. 3B). Therefore, it was possible that ZAP expression in HCMV infected cells was regulated by the type I interferon system and that differences in the ability of AD169 and Merlin(R1111) to control expression of interferon-regulated protein expression resulted in differences in ZAP expression in HCMV infected cells.

It has been reported that ZAP-S expression is stimulated by activation of the type I interferon signalling (17). To confirm that ZAP-S expression in our experiments was related to the activation of this system, we either stimulated cells with interferon-α (IFN-α) (Fig. 3C) or inhibited IFN-α signaling in HCMV infected cells with the Janus kinase (Jak) inhibitor ruxolitinib (Fig. 3D). Treatment of uninfected HFF cells with IFN-α resulted in notable expression of ZAP-S, but not ZAP-L (Fig. 3C). Activation of the IFN-α signaling pathway was demonstrated by expression of MxA in the presence of IFN-α (Fig. 3C). Conversely, ZAP-S expression diminished more over time in the presence of ruxolitinib, an inhibitor of the type I interferon signaling pathway, in both AD169 and Merlin(R1111) infected cells (Fig. 3D). However, even in the presence of ruxolitinib, ZAP-L and ZAP-S expression was induced, potentially due to IRF3-mediated activation of ZAP expression (43). Taken together, we found that ZAP-S expression in AD169 and Merlin(R1111) infected cells was partly dependent upon activation of the type I interferon signaling pathway.

Because IRF3 can control ZAP expression (43) and IRF-3 expression decreases over time in Merlin infected cells (42), we also investigated if IRF3 expression could affect ZAP expression upon HCMV infection (Fig. 3E). We infected HFF cells with AD169, Merlin(R1111) or Sendai virus, which can stimulate expression of IRF-3 regulated genes, and assayed expression of IRF-3 and IRF3-Ser396, a phosphorylation modification of IRF3 that is required for transcriptional transactivation. IRF3 expression was observed in all infections, but IRF3-Ser396 expression was detected only in cells infected with Sendai virus (Fig. 3E). This suggested that activated IRF3 was not required for ZAP expression in either AD169 or Merlin(R1111) infected cells.

A previous report found that mRNA encoding ZAP in Merlin(R1111) infected cells increased over 24 h.p.i. and fell to baseline levels by 72 hours post infection (41). Therefore, we investigated if differences in ZAP protein expression in HCMV infected cells were related to differences in expression of mRNA encoding ZAP. We used quantitative RT-PCR to analyze ZAP mRNA abundance (Fig. 3F). Infection of HFF cells with both AD169 and Merlin(R1111) resulted in an increase in ZAP mRNA at 24 h.p.i. followed by a decrease in mRNA expression over time, with no substantial differences in ZAP mRNA abundance between AD169 and Merlin(R1111). This implied that ZAP protein expression may have been regulated at a post-transcriptional level in HCMV infected cells.

We considered if post-translational modification by ubiquitin and subsequent proteasomal degradation was to account for loss of ZAP expression in Merlin(R1111), similar to ubiquitin mediated loss of other anti-HCMV factors in Merlin infected cells (41). Previous data generated using mass spectroscopy had indicated the presence of the proteasome inhibitor MG132 had no obvious effect on ZAP expression early in Merlin replication (before 24 h.p.i.) (41). However, the role of the proteasome had yet to be examined late in Merlin replication (from 48 h.p.i. onwards) had yet to be examined.

We compared HFF cells infected with Merlin(R1111) with infected cells treated with DMSO or MG132 at 48 hours post infection (Figs. 3G and 3H). No obvious difference in ZAP expression was found in the presence of DMSO compared to MG132 72 h.p.i, suggesting the proteasomal degradation of ZAP was not responsible for loss of ZAP expression in Merlin(R1111) infected cells. However, we note that DMSO treatment appeared to increase ZAP abundance in infected cells 72 h.p.i compared to untreated cells, indicating that the solvent for MG132 has an effect on protein levels.

### ZAP antiviral activity is moderately inhibited by the UL2-UL11 encoding region of HCMV Merlin

**Figure 4:**
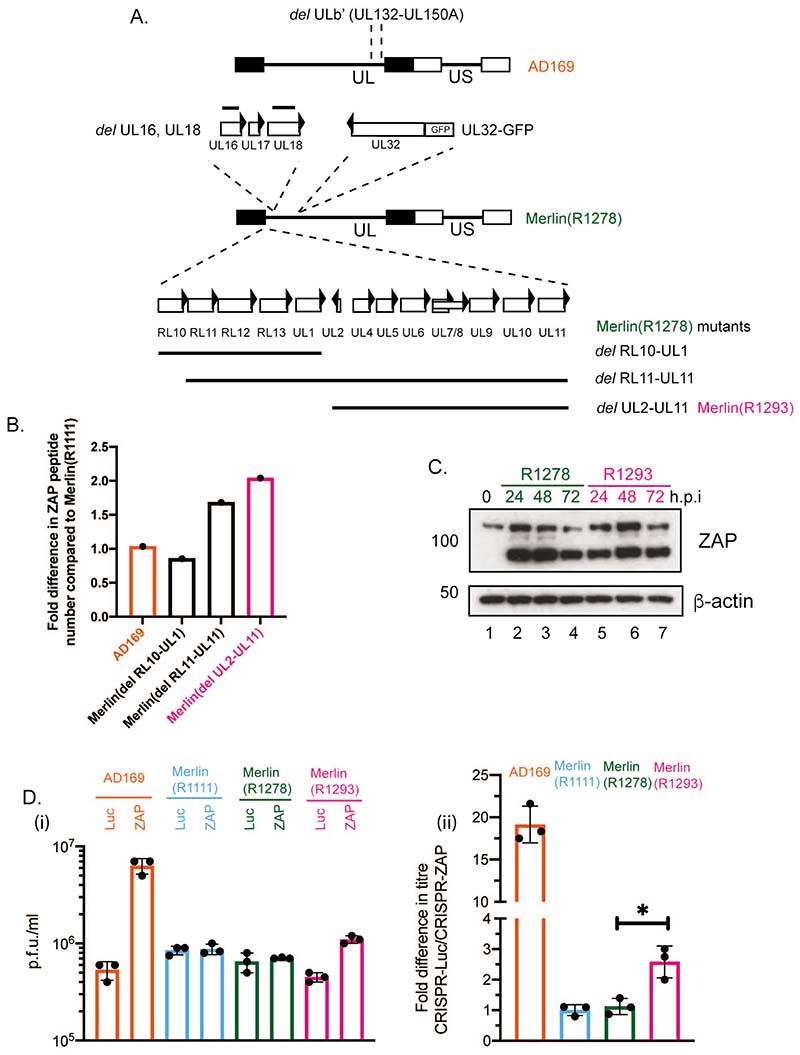
ZAP expression in HFF cells infected with Merlin mutants. (A) Schematic of the genomes of Merlin GFP reporter viruses. The major deletion in the AD169 genome is shown at the top (UL, unique long region; US, unique short region; rectangles, inverted repeat regions flanking UL and US). The relevant features of the Merlin(R1278) genome are indicated below AD169 and include the gene UL32 protein fused to GFP, the deletions in genes UL16 and UL18 (solid black lines). The deletions in three Merlin mutants, including Merlin (R1293), are indicated. (B) The number of peptides from ZAP proteins found at 72 hours post infection in cells infected with the viruses indicated in the figure. Data taken from reference (41). (C) HFF cells were infected with Merlin(R1278) or Merlin(R1293) at a multiplicity of infection of 1. Cell lysates were prepared for western blotting at 72 hours post infection. Uninfected cells harvested at the time of infection are shown as 0 h.p.i.. Proteins recognized by the antibodies used in each experiment are indicated to the right of each figure. The positions of molecular weight markers (kDa) are indicated to the left of each figure. Relative band intensity (band intensity relative to β-actin signal in the same lane) was analyzed using ImageJ and fold knockdown of ZAP-L at 72 h.p.i. is reported in the text. The data is representative of two independent experiments. (D) CRISPR cell lines were infected at a multiplicity of infection of 1 with viruses indicated in the figure. Virus in infected cell supernatant was collected at 96 hours post infection and viral titre was determined by titration of virus supernatant on HFF cells. Virus and cell line used is shown below each figure (i) Titre in plaque forming units/ml (p.f.u./ml) of each experiment. (ii) Data shown is the fold increase in virus titre in HFF cells containing CRISPR inhibiting ZAP expression compared to virus titre from HFF cells containing CRISPR inhibiting Luciferase expression. In each figure data is representative of three independent experiments (black data points) and presented as average (block) and standard deviation (error bars) of the data. Statistical relevance was examined used a student t test. ns = not significant (ns), p=<0.05 (*).

We then further considered how ZAP protein expression was regulated in Merlin(R1111) infected cells. A previous quantitative mass spectroscopy study (44) indicated that loss of the Merlin genomic regions encoding either RL11-UL11 or UL2-UL11, but not the region encoding either RL10-UL1, resulted in an increase in ZAP expression at 72 hours post infection (Fig. 4A and 4B). This suggested that proteins expressed in the UL2-UL11 region of Merlin could restrict ZAP expression and was consistent with the idea that that herpesviruses encode proteins that antagonize ZAP function (30, 31). There are notable difference in nucleotide sequences of the open reading frames encoding UL2-UL11 of the AD169, TB40/E and Merlin genomes (45), which could explain differences in the sensitivity of these strains to ZAP. Moreover, it was previously reported that ZAP could interact with RNAs from the HCMV genomic region that encodes protein UL4-UL6 (33), suggesting that HCMV RNA-protein interaction might influence ZAP expression.

We infected HFF cells with Merlin mutants that did (Merlin(R1278) or did not (Merlin(R1293) encode UL2-UL11 (Fig. 4A) and used western blotting to investigate ZAP expression. We observed a modest increase in ZAP-L in cells infected with HCMV Merlin lacking UL2-UL11 (3-fold increase in Merlin(R1293) (lane 7) compared to Merlin(R1278) (lane 4) in Fig. 4C). This data suggested that the presence of the Merlin genome encoding UL2-UL11 proteins could influence, albeit modestly, the expression of ZAP-L protein. Furthermore, when we examined titre of virus from CRISPR cells we observed a modest increase in HCMV Merlin replication in the absence of UL2-UL11 proteins (Merlin(R1293) in CRISPR-ZAP HFF cells compared to CRISPR-Luc HFF cells when compared to cells infected with the control virus Merlin(R1278) (Fig. 4D).

Therefore, a modest increase in ZAP-L expression in Merlin infected cells was associated with modest restriction of Merlin replication. However, restriction of Merlin in these experiments was not as great as restriction of AD169 (Fig. 4D), indicating that factors other than those in the UL2-UL11 region of Merlin could influence HCMV interaction with ZAP in Merlin infected cells.

### ZAP depletion has similar effects on HCMV AD169 and Merlin protein expression

**Figure 5:**
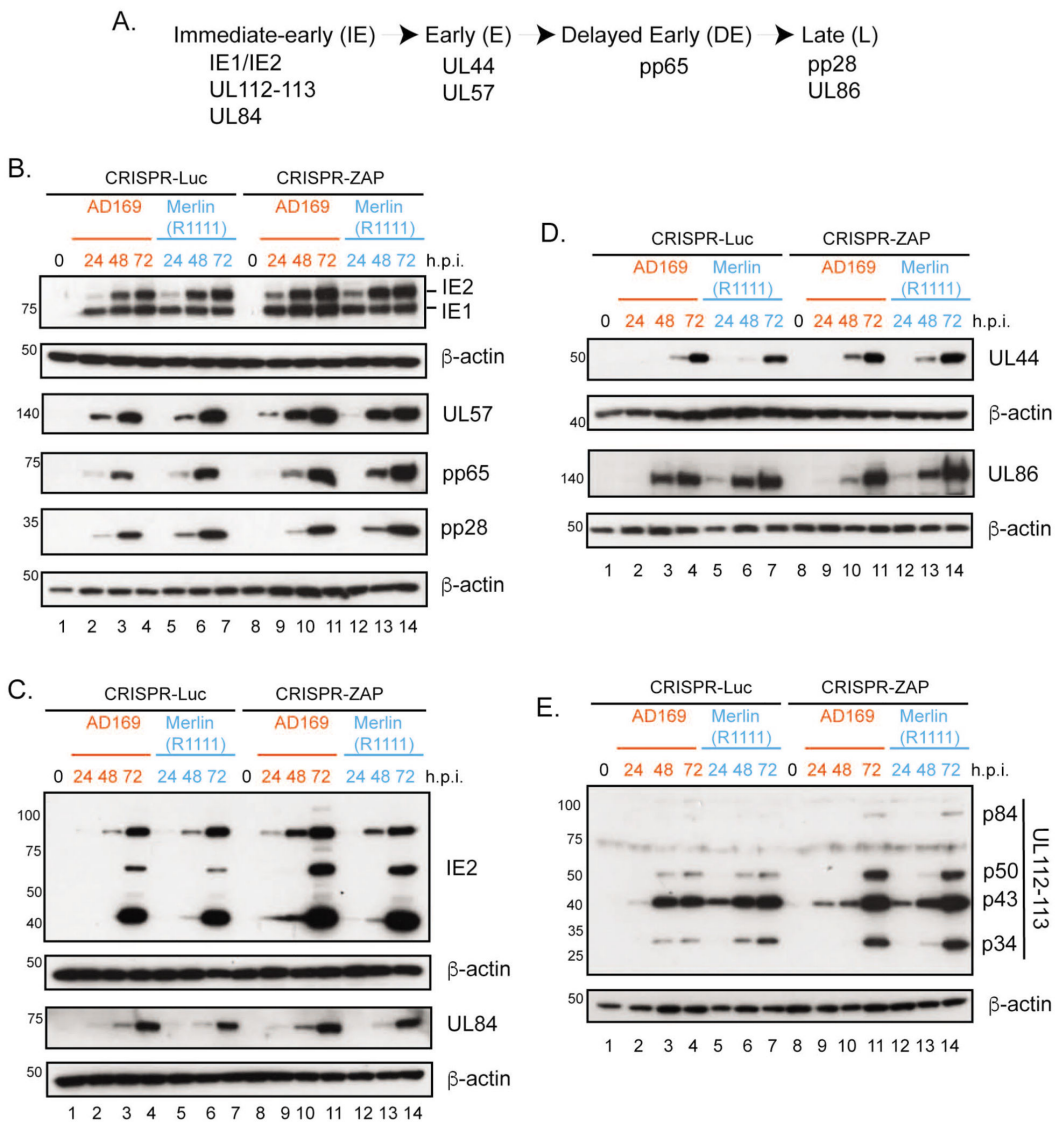
HCMV protein expression in infected cells. (A) Schematic of HCMV gene expression with relevant proteins grouped into kinetic classes. (B-E) HFF containing CRISPR inhibiting expression of either Luciferase (Luc) or ZAP were infected with either AD169 or Merlin(R1111) at a multiplicity of infection of 1. Cell lysates were prepared for western blotting at each time point indicated above the figure (hours post infection (h.p.i.)). Uninfected cells harvested at the time of infection are shown as 0 h.p.i.. Proteins recognized by the antibodies used in each experiment are indicated to the right of each figure. The positions of molecular weight markers (kDa) are indicated to the left of each figure. The data is representative of two independent experiments.

Previous studies have reported that ZAP-L and ZAP-S overexpression could influence expression of HCMV proteins such as IE2, UL44 and pp65 and quantitative mass spectrometry analysis of HCMV infected cells revealed an increase in some, but not all, HCMV proteins in ZAP knockdown cells (32, 33). Therefore, we compared expression of AD169 and Merlin(R1111) proteins from several stages of viral replication (Fig. 5A) in the CRISPR control and ZAP depleted cells shown in Figure 1. In both AD169 and Merlin(R1111) infected cells, we observed an increase in IE2, UL57 and pp65 expression in the absence of ZAP (Fig. 5B). We also found no obvious difference in IE1 or pp28 expression between AD169 and Merlin (1111) in the absence of ZAP (Fig. 5B). Crucially, we observed no difference in protein expression in AD169 and Merlin(R1111) infected cells that would account for the differences in HCMV infectious virus production observed in Figure 1.

We then analyzed IE2 expression, which is required for early and delayed early protein production, in greater detail. Expression of full length IE2 (IE2-86) is accompanied by expression of low molecular weight IE2 proteins (IE2-60 and IE2-40) from internal translational start codons in the mRNA expressing IE2-86. Using an antibody recognizing all IE2 proteins, we observed a that ZAP depletion led to increased expression of all IE2 proteins in both AD169 and Merlin(R1111) infected cells (Fig. 5C).

Expression of IE2-86 and IE2-40 results in post-translational stabilization of the essential early HCMV factor UL84, which has been reported as essential for early, delayed-early late HCMV gene expression, including expression of early protein UL44 and delayed-early protein pp65 (46). Therefore, increased expression of IE2 proteins could lead to increased expression of UL84, which could be related to increased expression of early or delayed-early proteins (Fig. 5A). However, we found that the increase in IE2 protein expression did not result in a large increase in an UL84 protein expression in ZAP CRISPR cells (Fig. 5C). Also, we found no obvious increase in UL44 expression between AD169 and Merlin(1111) in the absence of ZAP (Fig. 5D). Therefore, increased IE2 expression in the absence of ZAP in AD169 and Merlin(R1111) infected cells was not associated with a differential increase in either UL84 expression or production of proteins whose expression requires UL84.

We also investigated if there was any other functional relevance to the presence of increased IE2 protein expression in the absence of ZAP. IE2-86 is required for the transcriptional transactivation of the HCMV gene encoding UL112-113 proteins p84, p50, p43 and p34 (47), which should promote HCMV replication compartment formation and efficient genome replication (48). We found an increase in expression of all UL112-113 proteins in both AD169 and Merlin(R1111) infected cells (Fig. 5E). Thus, an increase in IE2 protein expression was associated with an increase in IE2 function that increased UL112-113 protein expression.

Overall, consistent with previous observations (32, 33), ZAP influenced the expression of several HCMV proteins in infected cells. The increase in IE2 expression in CRISPR-ZAP cells did not have wide-ranging effects on HCMV protein expression. Importantly, in all experiments there was no obvious difference in expression of any HCMV proteins between either AD169 and Merlin(R1111) infected cells, indicating differences in AD169 and Merlin(R1111) infectious virus production in the absence of ZAP were not related to differences in expression of the HCMV proteins examined here.

### The dinucleotide composition of HCMV mRNAs is similar between HCMV strains

To identify possible differences in protein expression between AD169 and Merlin(R1111), we examined the dinucleotide content of mRNAs expressed by both strains. Of note, it has been reported that addition of CpG dinucleotides to mRNA encoding HCMV IE resulted in decreased expression of IE1 in the presence of ZAP (32), indicating that ZAP could recognize an HCMV mRNA with a high CpG dinucleotide content.

**Figure 6:**
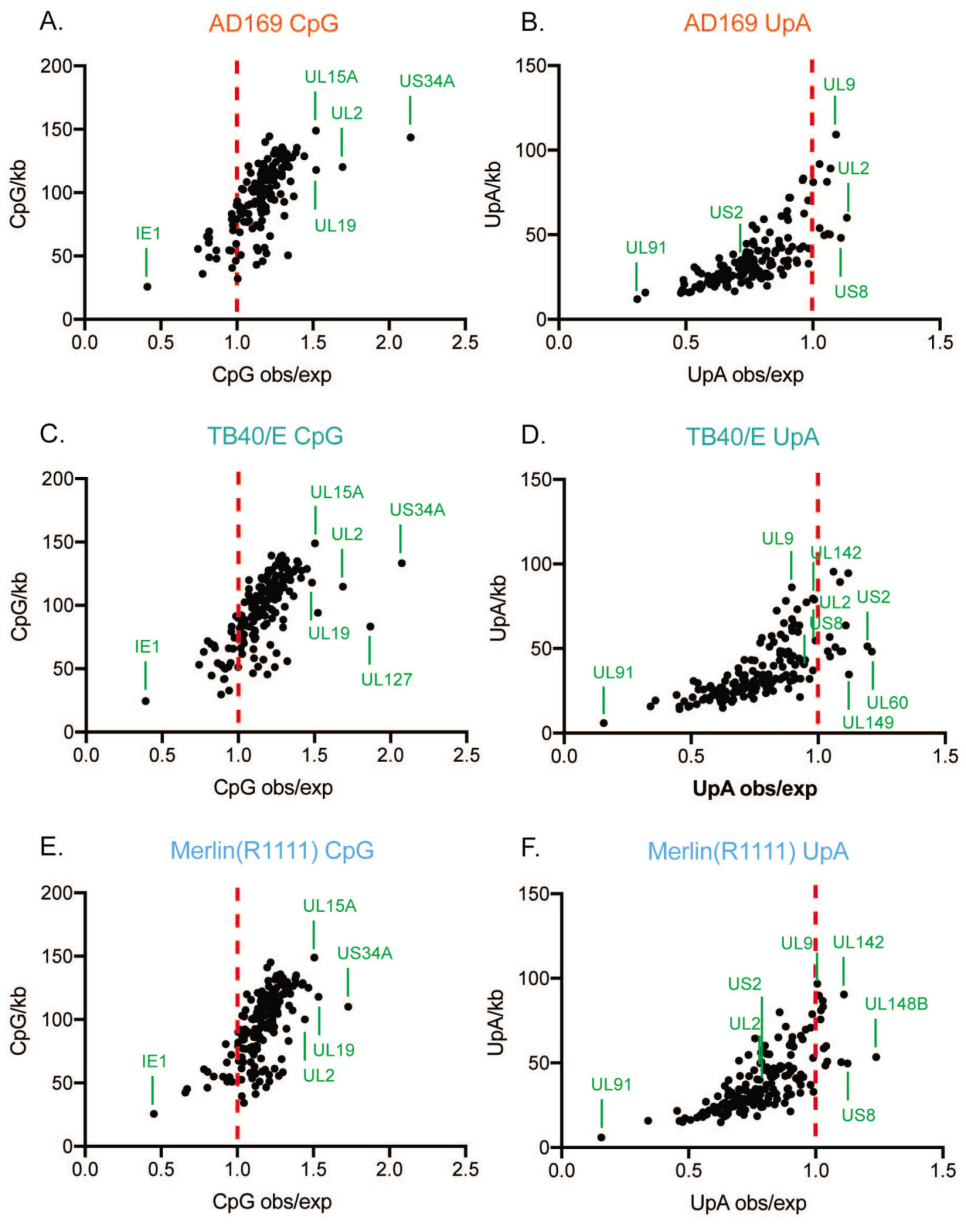
CpG and UpA dinucleotide content of AD169, Merlin and TB40/E mRNAs. The plots show observed verses expected (obs/exp) dinucleotide ratios in relation to the numbers of CpG or UpA dinucleotides per kilobase of RNA, with each dot representing an annotated viral RNA. The conditions are indicated at the top of each panel. The red dotted line in each figure indicates an obs/exp of 1. and the identities of selected outlying RNAs are marked. IE1 is indicated in (A), (C) and (E). In each figure the identities of the RNAs with the greatest CpG or UpA content are indicated.

We first sequenced the genomes of AD169 or Merlin(R1111) viruses used in our experiments and found no obvious differences in either genome sequence compared to previously published genome sequences (data not shown, see Material and Methods for information on sequence deposition). We then analyzed the mRNA sequences in each of our genomes and calculated the observed versus expected (obs/exp) ratio (6) of CpG content in each RNA (Fig. 6). This indicates whether CpG dinucleotides are over- or under-represented in any transcript analyzed in both genomes. An obs/exp ratio of 1 suggests that there is no over- or under-representation of CpG dinucleotide content in a nucleotide sequence. A similar analysis was conducted using TB40/E mRNA sequences deposited in GenBank.

Most of the mRNAs predicted to be produced by AD169, TB40/E and Merlin(R1111) had obs/exp ratios of between 1 and 1.5. This indicated that there was a modest overrepresentation of CpGs in RNAs produced by each HCMV strain (Figs. 6A, 6C and 6E), in contrast to most mammalian mRNA virus genomes (49, 50). Moreover, our analysis indicated that every mRNA produced by AD169 and TB40/E had similar obs/exp ratio to its Merlin(R1111) counterpart (Figs. 6A, 6C and 6E). Therefore, differences in CpG content of HCMV mRNAs did not appear to be responsible for differences in HCMV strain sensitivity to ZAP. Because ZAP has been reported to preferentially bind to a CpG dinucleotide within C(n7)GnCG motifs *in vitro* (5), we calculated the number of these sequences in AD169 and Merlin(R1111) mRNAs. We found no obvious difference in the abundance of this sequence between the strains (data not shown).

It has been reported that production of viral RNAs with high UpA dinucleotide content could also be inhibited by ZAP (9, 51). Therefore, we repeated our analysis, investigating the obs/exp ratios for the UpA dinucleotide content of RNAs produced by AD169, TB40/E and Merlin(R1111) (Figs. 6B, 6D and 6F). We found that nearly all mRNAs produced by all HCMV strains have scores of less than 1, indicating that UpA dinucleotides were underrepresented in most RNAs produced by all three HCMV virus strains. There were no consistent differences in the UpA dinucleotide content of the mRNAs produced from the different HCMV strains that correlated with their sensitivity to restriction by ZAP.

Additionally, we found no obvious relationship between protein expression in the presence and absence of ZAP (Fig. 5) and the CpG or UpA dinucleotide content of mRNAs. For example, IE2 and pp28 had near identical CpG and UpA content (data not shown), but IE2 protein expression was influenced by ZAP, whereas pp28 had only a very small change in expression (Fig. 5). Similarly, ZAP had been reported to bind several TB40 RNAs (UL4, UL5, UL6, UL50, UL75, UL92, UL102, UL132, US18, US27) (33). We found near identical CpG and UpA content of these mRNAs in AD169 compared to Merlin mRNAs (data not shown). These data further emphasized that there was no obvious relationship between the CpG and UpA content of HCMV mRNAs and the ability of ZAP to restrict replication of viruses producing those RNAs, supporting previous observations in HCMV and alphaviruses (33, 52).

### ZAP restricts production of both infectious cell-associated and cell-released HCMV

**Figure 7:**
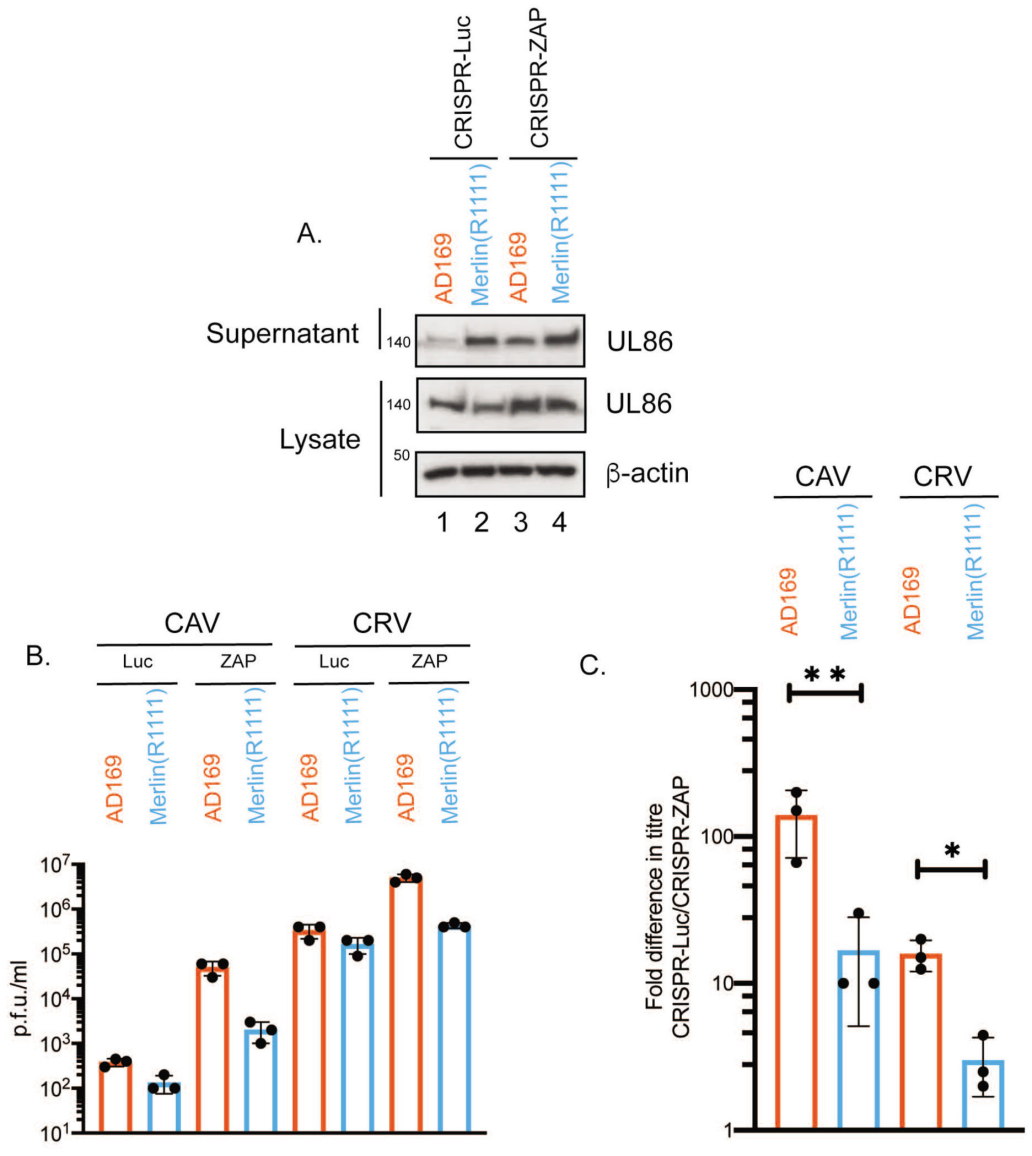
HCMV protein expression in CAV and CRV from HFF cells infected with AD169 or Merlin(R1111). (A) HFF containing CRISPR inhibiting expression of either Luciferase (Luc) or ZAP were infected with either AD169 or Merlin(R1111) at a multiplicity of infection of 1. At 96 hours post infection (h.p.i.) cell lysates were prepared for western blotting and cell supernatant was concentrated by centrifugation, then prepared for western blotting. Proteins recognized by the antibodies used in each experiment are indicated to the right of each figure. The positions of molecular weight markers (kDa) are indicated to the left of each figure. Relative band intensity (band intensity relative to β-actin signal in the same lane) was analyzed using ImageJ and fold of UL86 protein in supernatant is reported in the text. The data is representative of two independent experiments. (B) and (C) CRISPR cells were infected at a multiplicity of infection of 1. At 96 hours post infection virus in supernatant (cell release virus (CRV) was harvested and cell associated virus (CAV) was collected in tissue culture media by sonification. CRV and CAV titre was determined by titration of virus on HFF cells. Virus and cell line used is shown below each figure (B) Titre in plaque forming units/ml (p.f.u./ml) of each experiment. (C) Data shown is the fold increase in virus titre in each cell line compared to virus titre from HFF cells containing CRISPR inhibiting Luciferase expression. In each figure data is representative of three independent experiments (black data points) and presented as average (block) and standard deviation (error bars) of the data. Statistical relevance was examined used a student t test. p=<0.05 (*), p=<0.01 (**).

We found no obvious difference in viral protein expression that would account for differences in AD169 and Merlin(R1111) infectious virus production when ZAP was deleted. This included expression of the major capsid protein UL86 (Fig. 5D). However, in the supernatant from infected CRISPR-ZAP cells, there was a substantial increase in the amount of the virion-associated protein UL86 from AD169 (a 3-fold increase, compare lanes 1 and 3 in Fig. 7A), but not Merlin(R1111) (compare lanes 2 and 4 in Fig. 7A). Therefore, ZAP restricted the production of AD169 virions, whereas ZAP had no obvious effect on the production of Merlin(R1111) virions.

We then assayed how much infectious virus was found in infected cell supernatant (cell released virus (CRV)) and how much infectious virus remained associated with infected cells, ready to be released (cell associated virus (CAV)) in the presence and absence of ZAP (Figs. 7B and 7C). ZAP depletion increased both CRV and CAV produced from AD169 infected cells (Figs. 7B and 7C), suggesting that loss of ZAP in AD169 infected cells resulted in an enhanced production of infectious AD169 virus in infected cells that was subsequently released into the supernatant of AD169 infected cells. ZAP also had an effect on Merlin(R1111) CAV production (Figs. 7B and 7C), suggesting that this virus can be partially restricted by ZAP, though much less efficiently than AD169. Moreover, the increased production of both infectious CRV and CAV in the absence of ZAP for AD169 suggests that ZAP restricts a late stage of HCMV replication during production of infectious virus.

## Discussion

Previous reports have found that that ZAP could restrict replication of the HCMV strain TB40/E (32, 33). We confirmed this but also found that the magnitude of ZAP restriction for HCMV was dependent the strain analyzed. Briefly, we observed that production of the high passage HCMV strain AD169 was potently restricted by ZAP, whereas production of the clinical-like HCMV strain Merlin was resistant to this antiviral protein. The ability of Merlin, but not AD169, to evade inhibition of virus production was associated with reduced expression of ZAP expression in Merlin infected cells compared to AD169 infected cells 48 and 72 hours post infection. This may have been related to the ability of Merlin to effectively control the type I interferon response (42). Additionally, the differences in the ability of AD169 and Merlin(R1111) to evade ZAP did not appear to stem from an obvious difference in the CpG content of AD169 and Merlin mRNAs.

The ability of ZAP to restrict AD169 CRV production allowed us to make several observations about ZAP and its co-factors TRIM25 and KHNYN. We found that TRIM25 was not obviously required to inhibit AD169 replication. This may seem to contrast with a previous report which proposed TRIM25 restricted TB40/E replication (32). It is possible that TRIM25 restricts HCMV replication using virus strain- or cell type-dependent mechanisms. However, while it was shown that TRIM25 overexpression led to notable restriction of TB40/E production, TRIM25 depletion using siRNA resulted in only a very modest increase in TB40/E production, suggesting that our results do not substantially differ (32). This suggests that TRIM25 is an effective inhibitor of HCMV production only in cells with high levels of TRIM25. Whether physiological levels of type I interferon or other antiviral signaling pathways are sufficient to stimulate TRIM25 abundance so that it potently restricts viral replication remains unclear. Furthermore, we and others (32) found that TRIM25 depletion leads to increased ZAP-L expression, suggesting that TRIM25 regulated ZAP-L expression in uninfected and HCMV infected cells. However, in our experiments, this increase in ZAP-L expression was not associated with an obvious restriction of either AD169 or Merlin(R1111) production, suggesting that increased ZAP-L expression under these conditions did not increase its putative antiviral activity.

Our observations also indicate that the putative endoribonuclease KHNYN was involved in ZAP-mediated restriction of AD169 production. Little is known about KHNYN function, but it is required for ZAP-mediated restriction of viruses unrelated to HCMV, specifically HIV genomes with high CpG dinucleotide content and murine leukemia virus genomes (26). Therefore, working in concert with ZAP, KHNYN may be able to restrict replication of a diverse range of viruses, including retroviruses and herpesviruses.

Lower ZAP expression in Merlin(R1111) infected cells than AD169 infected cells is consistent with previous observations using by mass spectrometry that expression of a number of proteins with the ability to restrict HCMV decreases over time, including many stimulated by the type I interferon system (42). Decrease in expression of antiviral proteins controlled by the type I interferon system, including ZAP, was associated with a decrease in expression of several cellular proteins required for type I interferon signaling, including STAT2 (42). From this data and observations made herein, we propose that the ability of ZAP to influence AD169 and Merlin(R1111) replication may be due to differing abilities of the two HCMV strains to control the type I interferon signaling and, therefore, ZAP expression.

An outstanding question is what is recognized by ZAP in AD169 infected cells that leads to restriction of AD169 replication. A previous report using a plasmid expression system indicated that the presence of CpGs in the HCMV IE1 mRNA allowed ZAP to inhibit its expression (32). We found no consistent differences in the CpG dinucleotide content of AD169, TB40/E and Merlin(R1111) mRNAs. Therefore, strain-specific differences in CpG dinucleotide content of HCMV mRNAs may not determine whether ZAP can restrict HCMV replication. However, the CpG content of non-coding RNA was not examined in our study including mRNA untranslated regions (UTRs), long non-coding RNAs, and short non-coding RNAs. Non-coding regions in HCMV mRNAs have yet to be accurately mapped and it is unclear if all non-coding HCMV RNAs are expressed in both AD169 and Merlin(R1111) infected cells.

The lack of an obvious relationship between the CpG content of HCMV mRNAs and ZAP antiviral activity are consistent with observations made elsewhere. When TB40/E infected cells were examined using SLAM-seq and eCLIP (33), it was observed that ZAP interacted with viral mRNAs encoding UL4-UL6 and these transcripts were susceptible to ZAP-dependent degradation (33). However, the UL4-UL6 mRNAs did not have a high CpG dinucleotide content compared to other HCMV RNAs and it was suggested that ZAP binding to mRNA encoding UL4-UL6 may be due to the very high expression of transcripts from this region in HCMV infected cells (53), which might suggest that ZAP binding within that region was non-specific. It is also important to consider that TB40/E can influence the expression of cellular mRNAs in infected cells (33), although it is unknown what roles the proteins encoded by those mRNAs may have. It is possible that differences in the expression of cellular mRNAs account for differences in AD169 and Merlin(R1111) replication in the presence or absence of ZAP.

Our observations that HCMV can evade restriction by ZAP are consistent with reports that other herpesviruses evade restriction of replication by ZAP. HSV and MHV-68 evade ZAP restriction of replication by producing viral proteins that either degrade the mRNA encoding ZAP proteins or inhibit ZAP protein function by preventing ZAP-ZAP protein interaction (30, 31). We did not identify an HCMV encoded protein inhibitor of ZAP in this study. Therefore, it is possible herpesviruses can inhibit ZAP function either directly, via virus-encoded proteins, or indirectly, via manipulation of the type I interferon signaling that modulates the expression of ZAP.

Finally, we note that ZAP restriction of HCMV CAV production is reminiscent of ZAP restriction of the poxvirus vaccinia virus; wherein the presence of ZAP restricts production of infectious vaccinia virus within the infected cell, leading to the production of aberrant, dense, poxvirus particles in the infected cell (29). This may suggest that there is a mechanism of virus restriction controlled by ZAP that can potentially act on the late events in the replication cycle for both poxviruses strains and HCMV strains that are sensitive to restriction by ZAP. Cytoplasmic dense bodies are frequently observed in HCMV infected cells, but a relationship between these bodies, the production of CAV and ZAP function has yet to be described.

## Supplementary Material

Supplementary Data

## Importance

Determining the function of cellular antiviral factors can inform our understanding of virus replication. The zinc finger antiviral protein (ZAP) can inhibit the replication of diverse viruses. Here, we examined ZAP interaction with the DNA virus human cytomegalovirus (HCMV). We found HCMV strain-dependent differences in the ability of ZAP to influence HCMV replication, which may be related to interaction of HCMV strains with the type I interferon system. These observations impact upon our current understanding of how ZAP restricts HCMV and how HCMV interacts with the type I interferon system.
