# Supplementary material for: Strain-Dependent Restriction of Human Cytomegalovirus by Zinc Finger Antiviral Proteins
Source: J Virol. Author manuscript; Available in PMC 2023 Apr 5. (PMC10062169; doi:10.1128/jvi.01846-22)
Supplement: Supplementary Data [file EMS170712-supplement-Supplementary_Data.pdf]

Supplementary Data

Strain-Dependent Restriction of Human Cytomegalovirus by Zinc Finger Antiviral Proteins

Maria Jose Lista[a*], Adam A Witney[b*], Jenna Nichols[c], Andrew J Davison[c] Harry Wilson[a], Katie A Latham[b], Benjamin J Ravenhill[d], Katie Nightingale[d], Richard J Stanton[e], Michael P Weekes[d], Stuart J D Neil[a #], Chad M Swanson[a #] & Blair L Strang[b #]

Department of Infectious Diseases, School of Immunology & Microbial Sciences, King's College London, London, UK[a]; Institute of Infection & Immunity, St George's, University of London, London, UK[b]; MRC-University of Glasgow Centre for Virus Research, Glasgow, UK[c]; Cambridge Institute for Medical Research, School of Clinical Medicine, University of Cambridge, Cambridge, UK[d]; Division of Infection and Immunity, Cardiff University School of Medicine, Cardiff, UK[e]

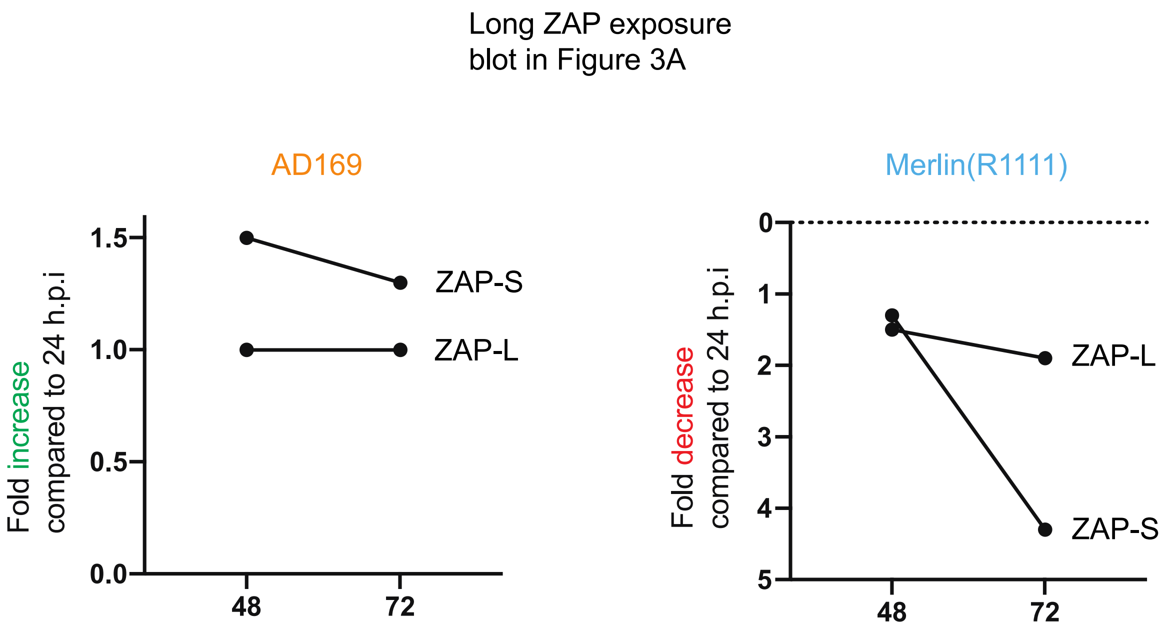

Figure S1

Figure S1 Quantification of ZAP expression. Relative band intensity (band intensity relative to β-actin signal in the same lane) of ZAP expression shown in Figure 3A (long exposure of ZAP blot) was analyzed using ImageJ. Data shown is fold difference in each protein expression at 48 and 72 h.p.i. compared to protein expression at 24 h.p.i.

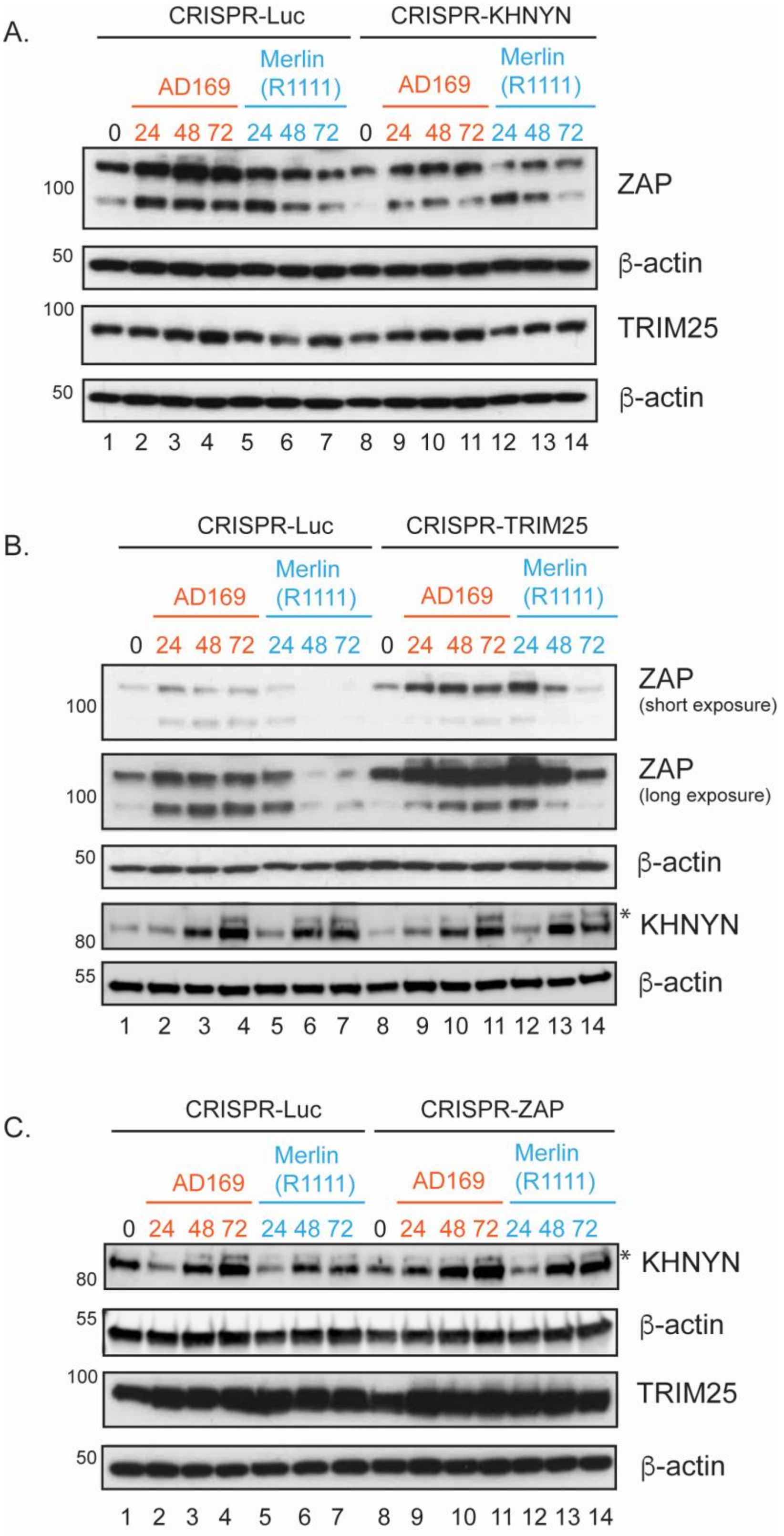
A.
CRISPR-Luc
CRISPR-KHNYN
AD169
Merlin (R1111)
0 24 48 72 24 48 72 0 24 48 72 24 48 72
ZAP
β-actin
TRIM25
β-actin
100
50
100
50
1 2 3 4 5 6 7 8 9 10 11 12 13 14
B.
CRISPR-Luc
CRISPR-TRIM25
AD169
Merlin (R1111)
0 24 48 72 24 48 72 0 24 48 72 24 48 72
ZAP (short exposure)
ZAP (long exposure)
β-actin
* KHNYN
β-actin
100
100
50
80
55
1 2 3 4 5 6 7 8 9 10 11 12 13 14
C.
CRISPR-Luc
CRISPR-ZAP
AD169
Merlin (R1111)
0 24 48 72 24 48 72 0 24 48 72 24 48 72
* KHNYN
β-actin
TRIM25
β-actin
80
55
100
50
1 2 3 4 5 6 7 8 9 10 11 12 13 14

Figure S2

Figure S2 Expression of ZAP, TRIM25 and KHNYN in HCMV infected cells. HFF cells treated with CRISPR (see Fig. 1) were infected at a multiplicity of infection of 1 with either AD169 or Merlin(R1111). Cell lysates were prepared for western blotting at the time points (hours post infection (h.p.i.)) indicated above the figure. Uninfected cells harvested at the time of infection are shown as 0 h.p.i.. Proteins recognized by the antibodies used in each experiment are indicated to the right of each figure. The positions of molecular weight markers (kDa) are indicated to the left of each figure. The data is representative of two independent experiments. High molecular weight KHNYN proteins referred to in the text are indicated with stars. A high molecular weight band recognized by the KHNYN antibody is indicated with a star in figures (A) and (C).
