# Supplementary material for: Strain-Dependent Restriction of Human Cytomegalovirus by Zinc Finger Antiviral Proteins
Source: J Virol. 2023 Mar 14;97(3):e01846-22. doi: 10.1128/jvi.01846-22 (PMC10062169; doi:10.1128/jvi.01846-22)
Supplement: Supplementary file 1 [file jvi.01846-22-s0001.pdf]

Supplementary Data

Strain-Dependent Restriction of Human Cytomegalovirus by Zinc Finger Antiviral Proteins

Maria Jose Lista[a*], Adam A Witney[b*], Jenna Nichols[c], Andrew J Davison[c] Harry Wilson[a], Katie A Latham[b], Benjamin J Ravenhill[d], Katie Nightingale[d], Richard J Stanton[e], Michael P Weekes[d], Stuart J D Neil[a #], Chad M Swanson[a #] & Blair L Strang[b #]

Department of Infectious Diseases, School of Immunology & Microbial Sciences, King's College London, London, UK[a]; Institute of Infection & Immunity, St George's, University of London, London, UK[b]; MRC-University of Glasgow Centre for Virus Research, Glasgow, UK[c]; Cambridge Institute for Medical Research, School of Clinical Medicine, University of Cambridge, Cambridge, UK[d]; Division of Infection and Immunity, Cardiff University School of Medicine, Cardiff, UK[e]

Long ZAP exposure
blot in Figure 3A

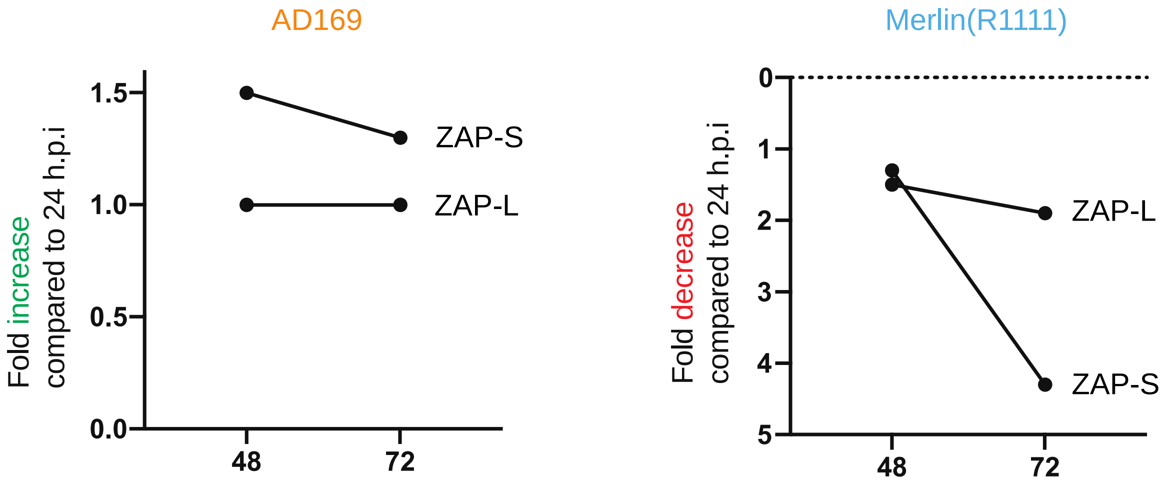

Figure S1

Figure S1 Quantification of ZAP expression. Relative band intensity (band intensity relative to β-actin signal in the same lane) of ZAP expression shown in Figure 3A (long exposure of ZAP blot) was analyzed using ImageJ. Data shown is fold difference in each protein expression at 48 and 72 h.p.i. compared to protein expression at 24 h.p.i.

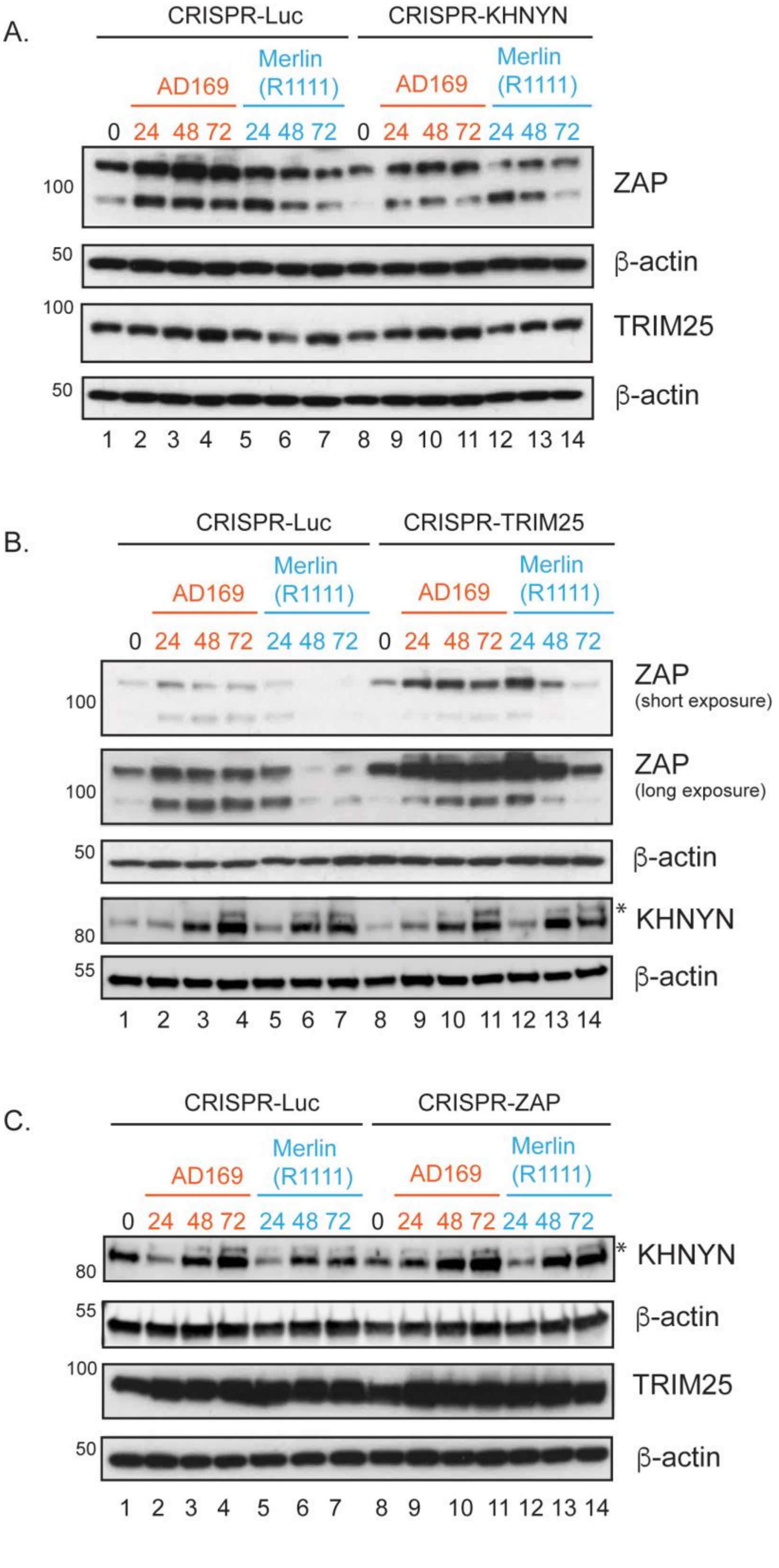
A.
CRISPR-Luc
CRISPR-KHNYN
AD169
Merlin (R1111)
0 24 48 72 24 48 72 0 24 48 72 24 48 72
100
ZAP
50
β-actin
100
TRIM25
50
β-actin
1 2 3 4 5 6 7 8 9 10 11 12 13 14
B.
CRISPR-Luc
CRISPR-TRIM25
AD169
Merlin (R1111)
0 24 48 72 24 48 72 0 24 48 72 24 48 72
100
ZAP (short exposure)
100
ZAP (long exposure)
50
β-actin
80
* KHNYN
55
β-actin
1 2 3 4 5 6 7 8 9 10 11 12 13 14
C.
CRISPR-Luc
CRISPR-ZAP
AD169
Merlin (R1111)
0 24 48 72 24 48 72 0 24 48 72 24 48 72
80
* KHNYN
55
β-actin
100
TRIM25
50
β-actin
1 2 3 4 5 6 7 8 9 10 11 12 13 14

Figure S2

Figure S2 Expression of ZAP, TRIM25 and KHNYN in HCMV infected cells. HFF cells treated with CRISPR (see Fig. 1) were infected at a multiplicity of infection of 1 with either AD169 or Merlin(R1111). Cell lysates were prepared for western blotting at the time points (hours post infection (h.p.i.)) indicated above the figure. Uninfected cells harvested at the time of infection are shown as 0 h.p.i.. Proteins recognized by the antibodies used in each experiment are indicated to the right of each figure. The positions of molecular weight markers (kDa) are indicated to the left of each figure. The data is representative of two independent experiments. High molecular weight KHNYN proteins referred to in the text are indicated with stars. A high molecular weight band recognized by the KHNYN antibody is indicated with a star in figures (A) and (C).
